# Admission Hyperglycemia as a Predictor of Mortality in Acute Heart Failure: Comparison between the Diabetics and Non-Diabetics

**DOI:** 10.3390/jcm9010149

**Published:** 2020-01-06

**Authors:** Jae Yeong Cho, Kye Hun Kim, Sang Eun Lee, Hyun-Jai Cho, Hae-Young Lee, Jin-Oh Choi, Eun-Seok Jeon, Min-Seok Kim, Jae-Joong Kim, Kyung-Kuk Hwang, Shung Chull Chae, Sang Hong Baek, Seok-Min Kang, Dong-Ju Choi, Byung-Su Yoo, Youngkeun Ahn, Hyun-Young Park, Myeong-Chan Cho, Byung-Hee Oh

**Affiliations:** 1 Department of Cardiovascular Medicine, Chonnam National University Medical School/Hospital, 42 Jebong-ro, Dong-gu, Gwangju 61469, Korea; jaeyeongcho@gmail.com (J.Y.C.);; 2 Division of Cardiology, University of Ulsan College of Medicine, Seoul 05505, Korea; 3 Department of Internal Medicine, Seoul National University Hospital, Seoul 03080, Korea; 4 Division of Cardiology, Sungkyunkwan University College of Medicine, Seoul 06351, Korea; 5 Department of Cardiology, Chungbuk National University College of Medicine, Cheongju 28644, Korea; 6 Department of Cardiology, Kyungpook National University College of Medicine, Daegu 41944, Korea; 7 Department of Cardiovascular Medicine, The Catholic University of Korea, Seoul 06591, Korea; 8 Department of Cardiology, Yonsei University College of Medicine, Seoul 03722, Korea; 9 Division of Cardiology, Seoul National University Bundang Hospital, Seongnam 13620, Korea; 10 Department of Internal Medicine, Yonsei University Wonju College of Medicine, Wonju 26426, Korea; 11 National Institute of Health (NIH), Osong 28159, Korea

**Keywords:** acute heart failure, hyperglycemia, diabetes mellitus, mortality

## Abstract

Background: To investigate the impact of admission hyperglycemia (HGL) on in-hospital death (IHD) and 1-year mortality in acute heart failure (AHF) patients with or without diabetes mellitus (DM). Methods: Among 5625 AHF patients enrolled in a nationwide registry, 5541 patients were divided into four groups based on the presence of admission HGL and diabetes mellitus (DM). Admission HGL was defined as admission glucose level > 200 mg/dL. IHD and 1-year mortality were compared. Results: IHD developed in 269 patients (4.9%), and 1-year death developed in 1220 patients (22.2%). DM was a significant predictor of 1-year death (24.8% in DM vs. 20.5% in non-DM, *p* < 0.001), but not for IHD. Interestingly, admission HGL was a significant predictor of both IHD (7.6% vs. 4.2%, *p* < 0.001) and 1-year death (26.2% vs. 21.3%, *p* = 0.001). Admission HGL was a significant predictor of IHD in both DM and non-DM group, whereas admission HGL was a significant predictor of 1-year death only in non-DM (27.8% vs. 19.9%, *p* = 0.003), but not in DM group. In multivariate analysis, admission HGL was an independent predictor of 1-year mortality in non-DM patients (HR 1.32, 95% CI 1.03–1.69, *p* = 0.030). Conclusion: Admission HGL was a significant predictor of IHD and 1-year death in patients with AHF, whereas DM was only a predictor of 1-year death. Admission HGL was an independent predictor of 1-year mortality in non-DM patients with AHF, but not in DM patients. Careful monitoring and intensive medical therapy should be considered in AHF patients with admission HGL, regardless of DM.

## 1. Introduction

Blood glucose level can be transiently elevated because of stress response to acute illness, so-called stress hyperglycemia (HGL). There is a growing body of evidence that admission HGL affects short- and long-term clinical outcomes in patients with acute myocardial infarction (AMI), regardless of the patients’ status of diabetes mellitus (DM) [1,2,3,4]. Microvascular dysfunction, proinflammatory status, and prothrombotic status in AMI patients with admission HGL might be possible explanations for these poor clinical outcomes. However, there is a paucity of data regarding the impact of admission HGL on clinical outcomes in acute heart failure (AHF).

Activation of the sympathetic nervous system (SNS) is one of major neuro-hormonal mechanism of the development or progression of HF. Decreased cardiac output activates SNS and thereby promotes myocyte hypertrophy and fibrosis, and in turn impairs diastolic and systolic function of both ventricles [5]. Activation of the SNS also causes inhibition of glucose-stimulated insulin secretion via the α-receptor. Pathological stress states may induce a metabolic state similar to diabetes with HGL and poor insulin responses to glucose challenge [6]. For these reasons, admission HGL can develop and reflect the degree or status of SNS activation in patients with AHF. If the higher admission glucose can reflect the higher SNS activation, therefore, it is presumed that admission HGL might be associated with clinical outcomes in patients with AHF. Actually, the study of Kattel S et al. [7] demonstrated that increased blood glucose level is a predictor of long-term mortality in patients with acute decompensated HF, irrespective of the DM status. Furthermore, in a study of 5428 previously non-DM patients with AHF, admission HGL was associated with higher in-hospital mortality than in AHF patients without admission HGL [8]. Contrary to results of these studies, however, admission glucose level was not a predictor of mortality a large cohort study of elderly HF patients [9]. Therefore, the association between admission glucose level and HF mortality should be clarified through larger studies.

The aim of this study, therefore, was to investigate the impact of admission HGL on clinical outcomes including in-hospital and long-term mortality in AHF patients with or without DM by analyzing the data from the nationwide large AHF cohort registry.

## 2. Materials and Methods

### 2.1. Study Population

A total of 5625 patients hospitalized for AHF from 10 tertiary university hospitals throughout the country were consecutively enrolled between March 2011 and February 2014 with a planned follow-up period through 2016. Patients who have signs or symptoms of HF and one of the following criteria are eligible for the study: (i) lung congestion or (ii) objective findings of left ventricular (LV) systolic dysfunction or structural heart disease. Lung congestion has been defined as ’congestion’ on a chest X-ray or as rales on physical examination. There are no exclusion criteria. The patients were classified into de novo (new-onset AHF in a patient without previously known cardiac dysfunction), acute decompensation of chronic HF, or five clinical profiles (acute decompensated HF, hypertensive HF, pulmonary edema, cardiogenic shock, and right HF) by the attending physician according to the 2005 European Society of Cardiology (ESC) guidelines [10]. The class of high-output HF was not recorded. The study protocol was approved by the ethics committee at each participating center, including Chonnam National University Hospital Institutional Review Board (project identification code: CNUH-2011-061). The present study was conducted according to the principles of the Declaration of Helsinki. All patients provided written informed consent for participation in the registry.

**Figure 1:**
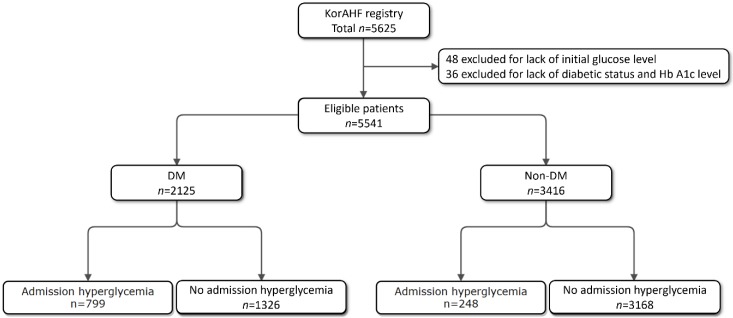
Study population. KorAHF, Korea Acute Heart Failure; DM, diabetes mellitus.

A total of 5625 cohort patients enrolled in Korean Acute Heart Failure (KorAHF) Registry. Of them, 48 patients were eliminated because they had no initial glucose level. Only AHF patients without history of DM and Hb. ≤ 6.4% were considered as non-DM patients in this study. Study population were divided into two groups according to the presence of DM; DM group (*n* = 2125, 70.4 ± 11.4 years) vs. non-DM group (*n* = 3416, 67.3 ± 16.0 years). Patients in each group were further divided into two groups according to the presence of admission HGL (admission serum glucose level > 200 mg/dL); HGL group (*n* = 248) vs. no HGL group (*n* = 3168) in non-DM; HGL group (*n* = 799) vs. no HGL group (*n* = 1326) in DM (Figure 1). In-hospital death (IHD) and 1-year death during clinical follow-up were compared. All-cause mortality between HF with preserved ejection fraction (HFpEF) and HF with reduced ejection fraction (HFrEF) were also compared in the presence or the absence of DM or HGL. HFrEF was defined as heart failure with initial ejection fraction of <40%.

### 2.2. Data Collection

Written informed consents were obtained from each patient. If patients were unable to give consent due to disease severity, informed consents were obtained from a relative or legal representative. The attending physician completed a web-based case report form in the Clinical Data Management System (iCReaT) from the Korea National Institute of Health (NIH) with the assistance of a clinical research co-ordinator. The detailed variables and values collected at baseline admission and case definition were described in elsewhere [11,12]. If the patients admitted via an emergency room, the initial presentation and laboratory results at emergency room were included in the baseline data. After discharge, events including all-cause death, death from HF aggravation, and re-hospitalization for HF aggravation were recorded. The latest information on a patient’s clinical manifestation, biochemistry, and medication is collected at the first re-visit in 30 days, and at 3, 6, 12, 24, 36, 48, and 60 months. The follow-up data were collected from the patients by the attending physician and stored in the web-based case report form. The outcome data for subjects who had not been followed up have been ascertained by a telephone interview. In addition, the outcome data for patients lost to follow-up were collected from the National Death Records.

### 2.3. Echocardiographic Measurements

Comprehensive echocardiographic studies including Doppler studies were performed according to the current recommendations of the American Society of Echocardiography. Left ventricular end-systolic and end-diastolic dimensions, interventricular septal and posterior wall thicknesses, and left atrial anteroposterior diameter were determined from two-dimensional images. EF was calculated using the conventional Teicholz’s and biplane Simpson’s method. Doppler echocardiograms were recorded on a strip chart recorder with a sweep speed of 100 mm/s. Early transmitral velocity (E wave) was measured using pulsed-wave Doppler from the apical four-chamber view, with the sample volume located at the tip of the mitral leaflets. Early diastolic (e’), late diastolic (a’), and systolic (s’) velocities at the septal mitral annulus were obtained in this view with tissue Doppler imaging. The E wave deceleration time (DT) was measured as the time between the peak early diastolic velocity and the point at which the steepest deceleration slope was extrapolated to the zero line.

### 2.4. Statistical Analysis

Continuous variables with normal distributions are presented as mean ± standard deviation and were compared using the Student’s *t*-test or Mann–Whitney *U* test when group distributions were skewed. Categorical variables were compared using the chi-square test or Fisher’s exact test, where appropriate. The comparison of baseline characteristics and echocardiographic findings between different sports discipline was performed using one-way analysis of variance. Cox proportional hazard regression was used to determine the independent effect of HGL in non-DM patients on long-term clinical outcomes. Variables with *p* < 0.1 on univariate regression analysis and clinically relevant variables were tested in the model. All statistical tests were two-tailed and *p* value < 0.05 were considered significant. All analyses were performed using the Statistical Package for Social Sciences, version 21.0 (SPSS, PC version, Chicago, IL, USA).

## 3. Results

### 3.1. Baseline Characteristics

Baseline characteristics are summarized in Table 1. DM was noticed in 2135 patients (38.6%); known DM in 1961 patients (92.3%) vs. newly diagnosed DM in 164 patients (7.7%). Admission HGL was noticed in 1047 patients (18.9%); 799 patients in DM group (37.6%) vs. 248 patients in non-DM group (7.3%). The overall comparison between DM vs. non-DM group was described in Supplementary Materials (Table S1).

In DM group, admission HGL was associated with more female sex, higher prevalence of pulmonary congestion, lower body mass index, higher blood pressure, higher heart rate, higher previous history of HF, higher prevalence of dyslipidemia and atrial fibrillation, and higher level of hemoglobin A1C, and high sensitivity C-reactive protein as compared to no admission HGL.

In non-DM group, admission HGL was associated with older age, higher prevalence of pulmonary congestion, lower body mass index, higher blood pressure, higher heart rate, more frequent previous history of HF, higher prevalence of hypertension, dyslipidemia, ischemic heart disease, and atrial fibrillation as compared to no admission HGL.

### 3.2. Echocardiographic Findings

Echocardiographic findings are summarized in Table 2.

In DM group, although LV ejection fraction was not different, LV and left atrial dimension were significantly larger in admission HGL group than in no admission HGL group. The parameters of diastolic function and systolic pulmonary artery pressure were not different between admission HGL and no admission HGL group.

In non-DM group, as in DM group, LV and left atrial dimension were also significantly larger in admission HGL group than in no admission HGL group even though LV ejection fraction was not different. Among non-DM patients, in contrary to DM group, E velocity, and E/E’ ratio were significantly higher in admission HGL group than in no admission HGL group.

### 3.3. Prescribed Medications

Angiotensin converting enzyme inhibitors or angiotensin receptor blockers were used similarly between the groups. However, beta-blockers were used more frequently used in diabetic patients with HGL than in those without HGL (59.1% vs. 50.5%, *p* < 0.001). Furosemide was used less frequently in non-diabetic patients with HGL (71.4% vs. 62.9%, *p* = 0.004). Digoxin was used more frequently in patients without HGL (18.4% vs. 25.2%, *p* < 0.001 in DM; 17.7% vs. 28.1%, *p* < 0.001 in non-DM) because atrial fibrillation was more prevalent in this patient group. Aspirin and statin were used more frequently in patients with HGL (58.3% vs. 51.9%, *p* = 0.004 in DM; 44.4% vs. 32.4%, *p* < 0.001 in non-DM)

#### Impacts of DM and Admission HGL on IHD and 1-Year Death

IHD developed in 269 patients (4.9%), and 1-year death developed in 1220 patients (22.2%).

Admission HGL was a significant predictor of both IHD (7.6% vs. 4.2%, *p* < 0.001) and 1-year death (26.2% vs. 21.3%, *p* = 0.001). DM was also a significant predictor of 1-year death (24.8% in DM vs. 20.5% in non-DM, *p* < 0.001), but DM was not a predictor of IHD.

**Figure 2:**
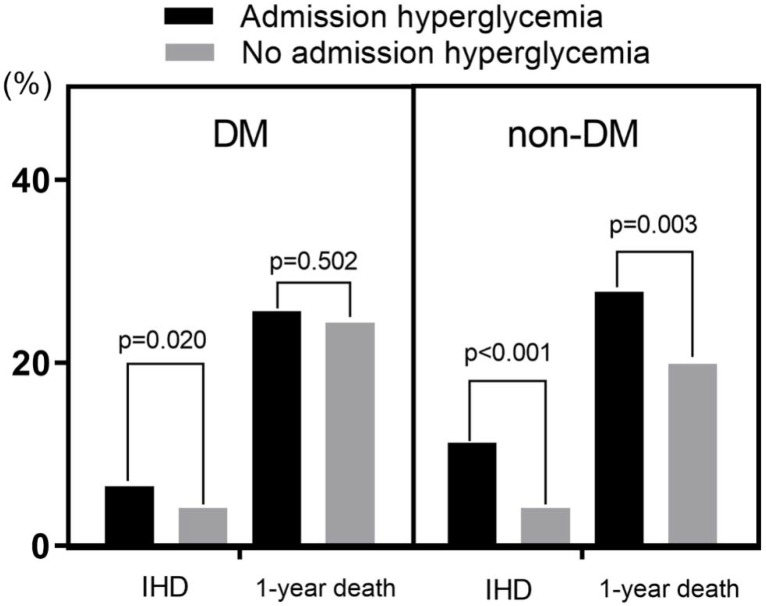
In-hospital death (IHD) and 1-year death according to the presence of diabetes mellitus (DM) and admission hyperglycemia. DM, diabetes mellitus; IHD, in-hospital death.

**Figure 3:**
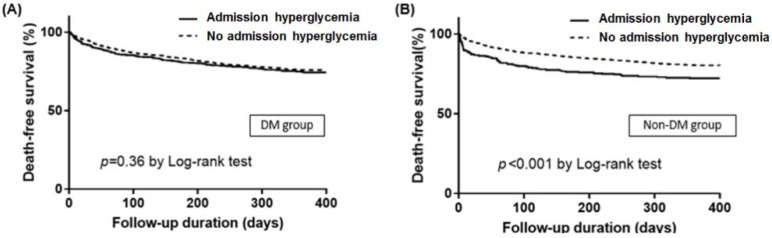
Kaplan–Meier survival curves for death free survival according to the presence of admission hyperglycemia in in patients with diabetes mellitus (DM) (**A**) and without diabetes (**B**) DM, diabetes mellitus.

In subgroup analysis, admission HGL was a significant predictor of IHD in both DM and non-DM group. Admission HGL was also a significant predictor of 1-year death in non-DM group (27.8% vs. 19.9%, *p* = 0.003), but it was not a predictor of 1-year death in DM group (Figure 2). In Kaplan–Meier survival curve analysis, admission HGL was associated with significantly lower death free survival in non-DM group, but not in DM group (Figure 3). In multivariate analysis, admission HGL was an independent predictor of IHD regardless of the presence of DM (Table 3), and admission HGL was also an independent predictor of 1-year mortality in non-DM patients (Table 4).

### 3.4. Impacts of DM and Admission HGL on 1-Year Mortality According to HF Subtypes

HFrEF was diagnosed in 3160 patients (59.7%), and HFpEF was diagnosed in 1269 patients (24.0%). HF with mid-range EF was diagnosed in 868 patients (16.3%).

**Figure 4:**
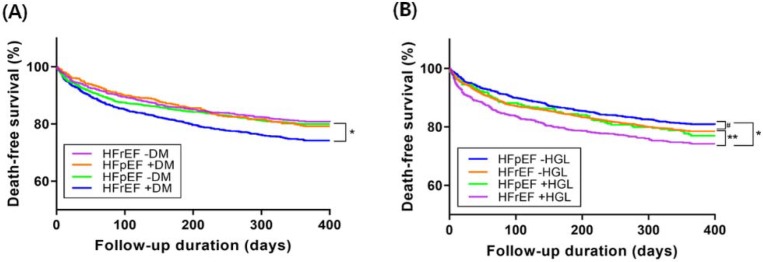
Kaplan–Meier survival curves for death-free survival according to the subtypes of heart failure, the presence of diabetes mellitus (DM) (**A**) and admission hyperglycemia (HGL) (**B**). HFrEF, heart failure with reduced ejection fraction; HFpEF, heart failure with preserved ejection fraction; DM, diabetes mellitus. *: *p* < 0.001, **: *p* < 0.01, #: *p* < 0.1 by log-rank test.

In Kaplan–Meier survival curve analysis, death free survival of HFrEF with DM group was the worst among the four patients and the death-free survival of other three groups were not so different one another (Figure 4A).

In Kaplan–Meier survival curve analysis, death free survival was significantly worse in HFrEF patients with admission HGL than in HFrEF patients without admission HGL or HFpEF patients. HFpEF patients without admission HGL showed trend toward better death-free survival than those HFpEF with admission HGL or HFrEF without admission HGL. Death-free survival was quite similar between HFpEF patients with admission HGL and HFrEF patients without admission HGL (Figure 4B).

## 4. Discussion

Through a nation-wide registry study for AHF, we investigated the impact of admission HGL on IHD and 1-year mortality in patients with or without DM, and the present study demonstrated several clinically important findings. Firstly, admission HGL was a significant predictor of IHD and 1-year death, whereas DM was only a predictor of 1-year death in patients with AHF and DM was not a predictor of IHD. Secondly, admission HGL was an independent predictor of 1-year mortality in non-DM patients with AHF, but not in DM patients. Thirdly, death free survival was significantly worse in HFrEF patients with DM than in HFrEF patients without DM or HFpEF patients, and death free survival was significantly worse in HFrEF patients with admission HGL than in HFrEF patients without admission HGL or HFpEF patients. To the best of our knowledge, the present study is the largest study evaluating the usefulness or clinical significance of admission HGL on IHD or 1-year mortality in patients with AHF according to the types of HF. In addition to the presence of DM, simple evaluation of admission HGL would be useful in identifying high-risk group of AHF or in the risk stratification of AHF. Careful monitoring and intensive medical therapy should be considered in AHF patients with admission HGL, regardless of DM.

### 4.1. Admission HGL or DM and IHD

In the present study, irrespective of DM, admission HGL was a significant predictor of IHD in AHF, but DM was not a predictor of IHD. Several studies also have shown that HGL is an independent predictor of IHD in patients with AHF [13,14,15,16]. Sud et al. also showed that mildly elevated presentation blood glucose is associated with 30-day mortality and hospitalization in AHF patients with no pre-existing DM [16]. Results from an international observation cohort showed that blood glucose concentrations at presentation are powerfully prognostic for 30-day mortality, independent of a diagnosis of DM [15]. Likewise, it was reported that stress HGL with AMI is associated with an increased risk of IHD in patients with and without DM [3]. Considering these results and our study, it is suggested that admission HGL is a simple but an important predictor of IHD in AHF. However, there are contradictory results regarding the impact of DM on IHD in patients with AHF. Some studies including large cohort study demonstrated that DM is an important predictor of IHD in AHF [17,18]. However, as in the result of our study, DM was not a predictor of IHD in other studies; rather, DM was an important predictor of mortality after hospital discharge from AHF [8,19]. Therefore, the issue regarding the impact of DM on IHD in patients with AHF should be clarified through further larger well-designed studies.

### 4.2. Admission HGL or DM and 1-Year Death

Basically, a few of the literatures so far reported about long-term effect of admission HGL. However, some reported that long-term outcome was also affected. Kattel et al. showed that 1.8 year-mortality was higher in ADHF patients with higher admission glucose level [7]. However, Aljohar et al. reported that HGL on admission is independently associated with hospital and short-term mortality in AHF patients [20]. In the contrary, Kosiborod et al. reported that they found no significant association between admission glucose levels and mortality in a large cohort of 50,532 elderly patients hospitalized with heart failure [9]. They said that outcomes seen in AMI cannot be readily applied to patients hospitalized with other cardiovascular conditions. Also, there were groups that reported that HGL in HF was a predictor of IHD but not of 1-year mortality [21,22]. Some group presented no effect on both short- and long-term mortality [23]. However, they performed a prospective study with relatively small subjects and used fasting glucose level, which is different from random admission glucose level. Despite of such a wide discrepancy between studies, however, the present study was unique in that hyperglycemic patients were divided into true diabetic and non-diabetic patients, showing that only non-diabetic patients with HGL had higher long-term mortality. Proposed mechanism is that HGL can increase cardiac contractility, which is only applied to patients with DM [24]. The authors reported slightly prolonged walking distance in type 2 diabetic heart failure patients in the study.

### 4.3. HGL and Increased SNS Affecting HF Mortality

The SNS is associated with glucose homeostasis by inhibiting insulin secretion and increasing glucagon secretion [25]. Therefore, HGL of non-DM patients may reflect stress state and increased SNS. However, HGL of DM patients is usually attributable to the poor glycemic control. There are reports that DM may be a strong independent predictor for HF. An animal study reported that DM causes metabolic shift from fat to carbohydrates and failure to increase myocardial glucose uptake in response to increased workload [26]. Another study showed that advanced heart failure causes myocardial insulin resistance by decreasing myocardial ATP levels and GLUT-4 translocation [27]. Consistent with that finding, there is growing evidence about myocardial insulin insensitivity in dilated cardiomyopathy (DCM). In heart failure patients with DCM, there is myocardial insulin insensitivity so that glucose is not used as fuel. In this situation, it is reported that HGL increases myocardial contractility [24]. Admission HGL may be the response to danger and reflect of activated SNS. However, treatment for diabetes might have the patient overcome this insensitivity. That is the proposed mechanism why HGL of diabetic patients did not affect long-term mortality. This has been also demonstrated in some clinical studies.

### 4.4. Impacts of DM and Admission HGL on 1-Year Mortality According to HF Subtypes

Usually, survival rate of HFpEF are better than that of HFrEF (*p* = 0.025 by log-rank test, Figure S1). In the present study, however, HFpEF and HFrEF showed no differences in 1-year mortality without DM (19.2% vs. 20.0%, *p* = 0.619). Patients with HFrEF showed higher mortality than those with HFpEF only in the presence of DM (25.7% vs. 20.8%, *p* = 0.022) in our data. However, MacDonald et al. [28]. showed that mortality benefit of HFpEF over HFrEF was maintained regardless of DM during the follow-up duration of 3.5 years. At 1-year follow-up, however, HFpEF with DM showed similar mortality with non-diabetic HFpEF, which is consistent with our study result. The mortality of non-diabetic HFrEF patients was higher than non-diabetic HFpEF patients, but this can be explained by dominant ischemic etiology of HFrEF in the study population.

There is paucity of data analyzing the impact of admission HGL according to HF subtypes. In the present study, HFpEF patients without HGL showed better survival than HFrEF patients with HGL (*p* < 0.001). In addition, they showed trend toward better survival than HFrEF patients without HGL and HFpEF patients with HGL. This may be translated into worse survival in patients with HGL regardless of LVEF, although the discriminative effect was greater in HFrEF. Therefore, it seems that HGL associated with SNS activation at presentation influence the long-term clinical outcome of patients with AHF. Indeed, there is a study that investigated the effect of nebivolol on HF in patients with or without DM [29]. The result showed that only non-DM group had benefit on primary outcome of all-cause mortality and cardiovascular hospitalization. Regulating SNS strategy such as adding beta-blockers in AHF patients with admission HGL might be warranted in future study.

## 5. Study Limitations

The present study has several limitations. First, as a prospective multicenter registry, not all variables that we want to analyze were collected. Second, there is no definite explanation for SNS activation and blood glucose except for proposed mechanism. However, this maybe hypothesis-generating at least. Third, rehospitalization rate was not analyzed in this study, since it was difficult to identify rehospitalization in the outside hospital. Therefore, the rehospitalization rate might not be precise. Fourth, there is no definite rule-out criteria using Hb A1c for patients with suspected DM. However, not all patients can undergo oral glucose tolerance test in the setting of AHF. Therefore, HbA1c could be a reasonable choice in this clinical situation. Finally, the 10 tertiary hospitals which participated in the present study might not be representative of the whole AHF population of Korea; however, as a relatively single-ethnicity country, 10 large-volume hospitals may represent the nation. There would be not too many differences.

## 6. Conclusions

Despite several potential limitations, the present study demonstrated that admission HGL, in addition to DM, is a simple and useful predictor of IHD and 1-year death in patients with AHF. Therefore, admission HGL can be useful in identifying high-risk group of AHF or in the risk stratification of AHF. Careful monitoring and intensive medical therapy for heart failure should be considered in AHF patients with admission HGL, regardless of the presence of DM. Further larger investigations to elucidate the actual mechanism for these results will be needed.

## Figures and Tables

**Table 1:** Baseline characteristics.

Variables	DM (*n* = 2125)			No DM (*n* = 3416)		
	Admission HGL (*n* = 799)	No Admission HGL (*n* = 1326)	*p*	Admission HGL (*n* = 248)	No Admission HGL (*n* = 3168)	*p*
Age (years)	70.8 ± 11.3	70.2 ± 11.5	0.223	72.9 ± 12.9	67.0 ± 16.1	<0.001
Male sex (%)	400 (50.1)	757 (57.1)	0.002	115 (46.4)	1670 (52.7)	0.054
Body mass index (kg/m^2^)	23.6 ± 3.6	24.0 ± 4.0	0.017	22.5 ± 3.3	23.0 ± 3.9	0.014
Systolic blood pressure (mmHg)	138.1 ± 33.0	132.1 ± 29.3	<0.001	139.0 ± 36.6	128.5 ± 29.1	<0.001
Diastolic blood pressure (mmHg)	79.9 ± 19.7	78.1 ± 17.6	0.035	82.3 ± 20.3	78.2 ± 18.8	0.003
Heart rate (bpm)	98.2 ± 26.9	90.4 ± 23.9	<0.001	102.3 ± 28.8	91.5 ± 26.0	<0.001
Hypertension	586 (73.3)	985 (74.3)	0.632	145 (58.5)	1564 (49.4)	0.006
Heart failure history	326 (40.8)	660 (49.8)	<0.001	84 (33.9)	1338 (42.2)	0.010
Dyslipidemia	645 (52.9)	443 (59.8)	0.003	97 (44.7)	984 (34.7)	0.003
Smoking history	304 (38.0)	550 (41.5)	0.118	99 (39.9)	1187 (37.5)	0.443
Alcohol history	278 (34.8)	502 (37.9)	0.156	78 (31.5)	1264 (39.9)	0.009
Valvular heart disease	72 (9.0)	165 (12.4)	0.015	21 (8.5)	542 (17.1)	<0.001
Cerebrovascular disease	139 (17.4)	253 (19.1)	0.333	34 (13.7)	414 (13.1)	0.775
Ischemic heart disease	304 (38.0)	535 (40.3)	0.294	68 (27.4)	654 (20.7)	0.012
Chronic kidney disease	169 (21.2)	302 (22.8)	0.383	19 (7.7)	304 (9.6)	0.316
Atrial fibrillation	145 (18.1)	392 (29.6)	<0.001	54 (21.8)	941 (29.7)	0.008
Serum glucose (mg/dL)	291.1 ± 87.3	137.1 ± 36.0	<0.001	259.1 ± 61.2	120.8 ± 27.8	<0.001
Hb A1c (%)	8.0 ± 1.6	6.9 ± 1.0	<0.001	5.8 ± 0.3	5.8 ± 0.4	0.764
Serum creatinine (mg/dL)	1.8 ± 1.6	1.7 ± 1.6	0.373	1.5 ± 1.4	1.3 ± 1.4	0.132
Glomerular filtration rate (ml/min)	61.0 ± 36.1	66.0 ± 39.9	0.004	67.1 ± 33.7	79.6 ± 40.0	<0.001
High-sensitivity C-reactive protein*	1.02 (0.26–3.71)	0.67 (0.23–2.24)	<0.001	0.88 (0.24–2.41)	0.58 (0.18–2.00)	0.684
Brain natriuretic peptide*	993.8 (617.3–1845.5)	893.3 (470.0–1685.8)	0.213	1004.4 (521.5–2120.0)	884.3 (447.1–1732.1)	0.188
N-terminal Pro-B type natriuretic peptide*	6198.3 (2441.5–15,835.3)	5365.0 (2344.0–13,443.0)	0.186	5983.0 (2361.0–13,833.0)	4497.0 (2031.0–10,416.0)	0.166
Troponin-I*	0.13 (0.05–1.20)	0.06 (0.03–0.21)	0.189	0.11 (0.05–0.76)	0.05 (0.03–0.19)	0.301

DM, diabetes mellitus; HGL, hyperglycemia. * presented as median value (interquartile range).

**Table 2:** Echocardiographic findings.

Variables	DM (*n* = 2125)			No DM (*n* = 3416)		
	Admission HGL (*n* = 799)	No Admission HGL (*n* = 1326)	*p*	Admission HGL (*n* = 248)	No Admission HGL (*n* = 3168)	*p*
LVEDD (mm)	56.2 ± 8.9	57.9 ± 9.2	<0.001	55.9 ± 9.7	57.5 ± 10.7	0.018
LVESD (mm)	44.1 ± 11.0	45.8 ± 11.6	0.001	43.5 ± 11.3	45.1 ± 13.0	0.047
LVEF (%)	36.8 ± 14.5	36.8 ± 15.1	0.996	37.0 ± 14.5	38.7 ± 16.1	0.081
LA dimension (mm)	45.7 ± 8.3	48.7 ± 9.0	<0.001	45.8 ± 10.6	48.7 ± 10.3	<0.001
E (m/s)	0.93 ± 0.35	0.96 ± 0.36	0.156	0.82 ± 0.33	0.95 ± 0.41	<0.001
DT (msec)	162.4 ± 59.5	167.5 ± 70.7	0.157	171.3 ± 69.7	172.1 ± 89.9	0.900
E’ (cm/s)	4.8 ± 3.4	4.7 ± 1.9	0.746	4.7 ± 1.9	5.2 ± 2.2	0.003
S’ (cm/s)	4.8 ± 1.8	5.1 ± 1.9	0.020	5.2 ± 2.0	5.1 ± 2.1	0.835
E/E’	22.3 ± 11.0	22.8 ± 12.6	0.401	19.1 ± 8.5	20.5 ± 11.3	0.039
SPAP (mmHg)	44.2 ± 14.6	45.2 ± 15.5	0.237	41.2 ± 14.3	43.5 ± 15.0	0.056

DM, diabetes mellitus; HGL, hyperglycemia; LVEDD, left ventricular end-diastolic dimension; LVESD, left ventricular end-systolic dimension; LVEF, left ventricular ejection fraction; LA, left atrium; E, early diastolic velocity of mitral inflow; DT, deceleration time of mitral inflow; E’, early diastolic velocity of septal mitral annulus; S’, systolic velocity of septal mitral annulus; RVSP, systolic pulmonary artery pressure.

**Table 3:** Independent predictors for in-hospital death in patients with and without diabetes mellitus

Variables	DM (*n* = 2125)			Non-DM (*n* = 3416)		
	Odd Ratio	95% CI	*p*	Odd Ratio	95% CI	*p*
Age > 75 years	1.72	1.07–2.79	0.026	0.92	0.65–1.30	0.625
Male sex	1.08	0.67–1.75	0.739	1.43	1.02–2.01	0.036
History of hypertension	0.98	0.59–1.62	0.927	0.94	0.67–1.33	0.740
Systolic blood pressure < 100 mmHg	3.59	2.17–5.93	<0.001	2.88	2.06–4.05	<0.001
Renal insufficiency (initial GFR < 60 mL/min)	1.70	1.05–2.76	0.030	2.14	1.53–2.99	<0.001
Admission hyperglycemia	1.84	1.11–3.05	0.017	2.26	1.47–3.48	<0.001
Hb A1c	0.83	0.68–1.02	0.080			
Ischemic etiology	1.88	1.16–3.04	0.010	1.91	1.37–2.68	0.74

DM, diabetes mellitus; CI, confidence interval; GFR, glomerular filtration rate.

**Table 4:** Independent predictors for 1-year death in patients without diabetes mellitus.

Variables	Hazard Ratio	95% CI	*p*
Age > 75 years	1.94	1.65–2.29	<0.001
Male sex	1.41	1.21–1.64	<0.001
Systolic blood pressure < 100 mmHg	1.84	1.54–2.20	<0.001
Renal insufficiency (initial GFR < 60 mL/min)	1.82	1.55–2.13	<0.001
Admission HGL	1.32	1.03–1.69	0.030
No RAS inhibitors	2.13	1.78–2.55	<0.001

CI, confidence interval; GFR, glomerular filtration rate; HGL, hyperglycemia; RAS, renin-angiotensin system.

## Supplementary Materials

The following are available online at https://www.mdpi.com/2077-0383/9/1/149/s1, Table S1: Comparison of baseline characteristics between DM and non-DM. Figure S1: Comparison of death-free survival between patients with heart failure with preserved versus reduced ejection fraction. HFpEF, heart failure with preserved ejection fraction; HFrEF, heart failure with reduced ejection fraction.
