# Supplementary material for: Admission Hyperglycemia as a Predictor of Mortality in Acute Heart Failure: Comparison between the Diabetics and Non-Diabetics
Source: J Clin Med. 2020 Jan 6;9(1):149. doi: 10.3390/jcm9010149 (PMC7019900; doi:10.3390/jcm9010149)
Supplement: Supplementary file 1 [file jcm-09-00149-s001.pdf]

Supplementary Table. Comparison of baseline characteristics between DM and non-DM.

Variables	DM (n=2,125)	Non-DM (n=3,416)	p
Age (years)	70.4±11.4	67.4±15.9	<0.001
Male sex (n, %)	1157 (54.4)	1785 (52.3)	0.112
Body mass index (kg/m2)	23.9±3.9	23.0±3.9	<0.001
Hypertension	1571 (73.9)	1709 (50.0)	<0.001
Diabetes mellitus history	1961 (92.3)	0 (0.0)	<0.001
Dyslipidemia	1088 (55.5)	1081 (35.4)	<0.001
Heart failure history	986 (46.4)	1422 (41.6)	0.001
Smoking history	854 (40.2)	1286 (37.6)	0.059
Alcohol history	780 (36.7)	1342 (39.3)	0.055
Obesity	133 (6.3)	145 (4.3)	0.001
Ischemic heart disease	839 (39.5)	722 (21.1)	<0.001
Cerebrovascular disease	392 (18.4)	448 (13.1)	<0.001
Chronic kidney disease	471 (22.2)	323 (9.5)	<0.001
Chronic obstructive lung disease	234 (11.0)	390 (11.4)	0.647
Atrial fibrillation	537 (25.3)	995 (29.1)	0.002
Malignancy	154 (7.2)	300 (8.8)	0.043
Systolic blood pressure (mmHg)	134.3±30.8	129.2±29.8	<0.001
Diastolic blood pressure (mmHg)	78.8±18.5	78.5±18.9	0.572
Heart rate (bpm)	93.3±25.3	92.3±26.3	0.159
Hb A1c (%)	7.3±1.4	5.8±0.4	<0.001
N-terminal Pro-B type natriuretic peptide (pg/ml)	10213.3±10970.8	8672.5±10681.0	<0.001
Brain natriuretic peptide (pg/ml)	1392.8±1378.2	1305.0±1256.2	0.124
High-sensitivity C-reactive protein (mg/dL)	2.6±4.6	2.1±3.9	0.002
Troponin-I (ng/mL)	3.0±15.7	2.8±22.3	0.722

Values are mean±SD or n (%). DM, diabetes mellitus.

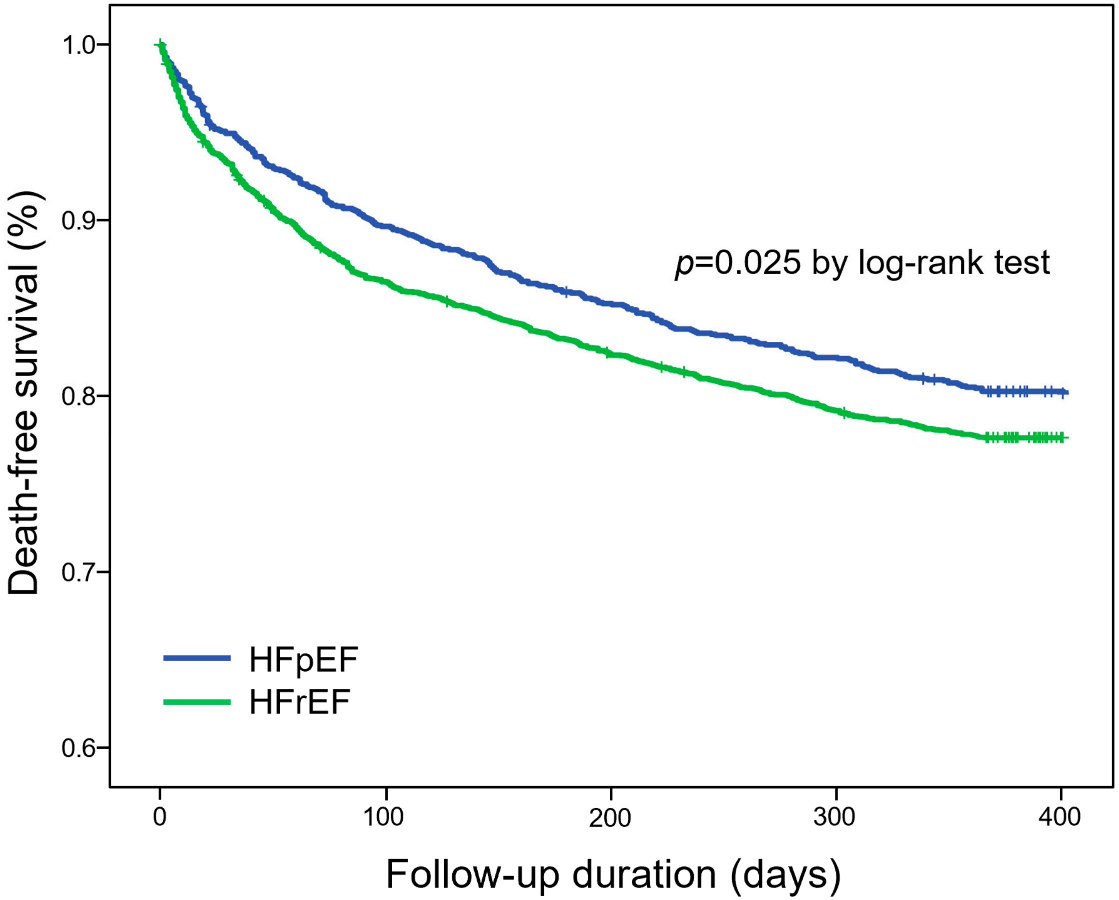
1.0
0.9
0.8
0.7
0.6
Death-free survival (%)
p=0.025 by log-rank test
HFpEF
HFrEF
0
100
200
300
400
Follow-up duration (days)

Figure S1
